# Interpretable and reproducible machine learning model for coronary calcification and segment-level stenoses stratification on computed tomography angiography

**DOI:** 10.1186/s12916-025-04478-0

**Published:** 2025-11-26

**Authors:** Jian Chen, Hongqiu Wang, Yiran Wei, Yu Xu, Guangming Wang, Yonghao Li, Zeyu Gao, Kaixuan Li, Xiaowei Zhou, Jin Zheng, Ziming Wang, Yuan Huang, Zhongzhao Teng, James H. F. Rudd, Lorena Escudero Sánchez, Michelle C. Williams, David E. Newby, Jonathan R. Weir-McCall

**Affiliations:** 1 Department of Radiology, School of Clinical Medicine, University of Cambridge, Cambridge, UK; 2 Department of Systems Hub, Hong Kong University of Science and Technology (Guangzhou), Guangzhou, China; 3 School of Biomedical Engineering & Imaging Science, King’s College London, London, UK; 4 British Heart Foundation Cardiovascular Epidemiology Unit, Department of Public Health and Primary Care, University of Cambridge, Cambridge, UK; 5 Cambridge Baker Systems Genomics Initiative, Department of Public Health and Primary Care, University of Cambridge, Cambridge, UK; 6 Department of Engineering, University of Cambridge, Cambridge, UK; 7 Department of Clinical Neuroscience, School of Clinical Medicine, University of Cambridge, Cambridge, UK; 8 Department of Oncology, University of Cambridge, Cambridge, UK; 9 Department of Cardiac Surgery, Xiangya Hospital, Central South University, Changsha, China; 10 School of Life and Health Sciences, Fujian Fuyao University of Science and Technology, Fuzhou, China; 11 Nanjing Jingsan Medical Science and Technology, Ltd., Jiangsu, China; 12 Department of Medicine, University of Cambridge, Cambridge, UK; 13 BHF Centre for Cardiovascular Science, University of Edinburgh, Edinburgh, UK; 14 Department of Radiology, Royal Papworth Hospital, Cambridge, UK

**Keywords:** Computed tomography angiography, Stable feature, Calcification quantification, Major coronary segments, Stenoses assessment, Interpretable machine learning model

## Abstract

**Background:**

Coronary computed tomography angiography (CCTA) is widely used as a first-line tool for diagnosing and managing coronary artery disease (CAD), and machine learning (ML)-based analysis shows promise for quantitative CAD assessment.

**Methods:**

In this post hoc analysis of 909 participants from the SCOT-HEART trial (median follow-up, 5.8 years), we first evaluated the distribution of CCTA-derived imaging features in a cohort (*n* = 221) with a zero calcium score, stenoses < 10%, and no evidence of CAD on CCTA, across 21 image processing settings. Interpretable ML models were then developed and validated to quantify coronary calcification and stenoses in major coronary segments (LMA, LCX, LAD, pRCA, mRCA). Calcified plaques, stenoses, and myocardial infarction outcomes were comprehensively assessed.

**Results:**

A total of 549 stable imaging features was identified across processing settings. Six ML algorithms (SVM, KNN, MLP, Naïve Bayes, gradient boosting, LightGBM) were evaluated for predicting coronary calcification and stenoses. The best model achieved an accuracy of 84.2% and an AUC of 0.973. Stenosis stratification accuracy exceeded 84.8% across all segments, with minimal (< 0.05) differences between models using all versus stable features. SHAP analysis indicated heterogeneous contributions of imaging phenotypes and clinical risk factors.

**Conclusions:**

Stable imaging features provide a reference for future ML-based coronary quantitatively assessments. Interpretable ML models demonstrated promising performance in quantifying coronary calcification and segment-level stenoses.

**Graphical Abstract:**

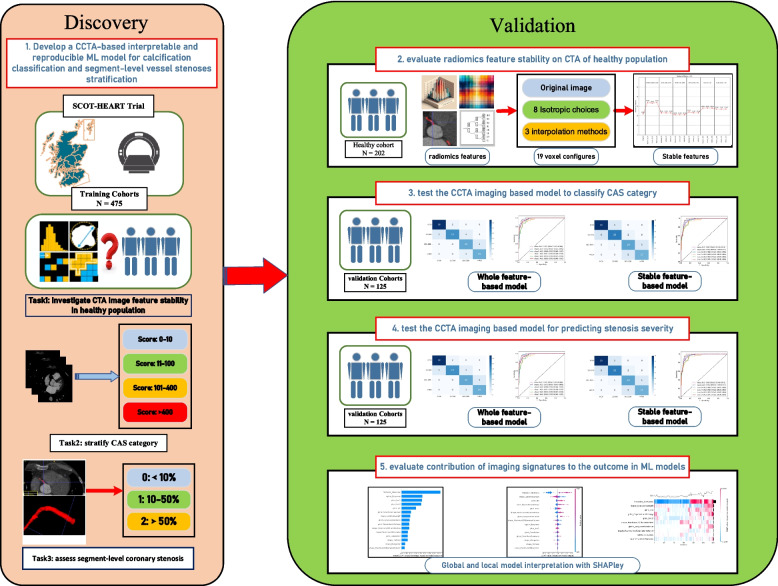

**Supplementary Information:**

The online version contains supplementary material available at 10.1186/s12916-025-04478-0.

## Highlights

1. Investigated the distribution and stability of pericoronary imaging phenotypes in populations without CAD using a median of 5.8-year follow-up multicentre datasets.

2. Conducted a machine learning-based analysis of coronary CTA and clinical risk factors to quantitatively evaluate outcomes in patients with coronary artery disease.

3. Explored the contributions of vascular risk factors and CTA imaging phenotypes in six kinds of predictive models for coronary artery disease outcomes.

**Supplementary Information:**

The online version contains supplementary material available at 10.1186/s12916-025-04478-0.

## Background

Cardiovascular disease including coronary artery disease (CAD) is the leading cause of global mortality and a significant contributor to disability worldwide [1, 2]. The primary process underpinning the majority of CAD is atherosclerosis [3]. Coronary computed tomography angiography (CCTA) provides an ideal means to assess atherosclerosis, providing an accurate noninvasive means of assessing both plaque and stenosis [4]. Several studies have shown the utility of plaque quantification and pericoronary fat attenuation in risk prediction, adding to traditional cardiovascular risk factors [5, 6]. While powerful biomarkers, these techniques do not capture the full granularity of the data held within the CCTA images. Image-based radiomics provide assessments which combined with machine learning algorithms hold promise to quantify and evaluate biomarkers, such as plaques. Plaque radiomics has been used to better characterize high-risk plaques [7, 8], while radiomics of the pericoronary fat has been used to discern oedematous versus fibrotic changes in the fat and as a prognostic marker for major cardiovascular events [9].

The literature reveals two characteristics to highlight current machine learning-based studies on CAD. Firstly, most existing studies on the automatic evaluation of coronary artery stenosis or calcification rely on deep-learning algorithms that perform end-to-end processes for feature extraction, dimensionality reduction, and modelling, often functioning as a non-interpretable tool. Concerns raised by medical experts regarding the application of AI in medicine—such as feature stability and model interpretability—are also evident in the subfield of AI application for cardiovascular imaging [10]. Secondly, most machine learning research focuses on assessing stenosis at the patient level, with limited attention to segment-level evaluations. However, segmental assessments of coronary artery stenosis are more aligned with precision medicine and personalized diagnostics. CAD diagnosis depends on segment-specific factors, including the number of affected arteries, lesion location and severity, and the length and tortuosity of narrowed segments, making segment-level analysis crucial for accurate diagnosis and prognosis.

Standardization is a critical factor in ensuring the reproducibility of machine learning models in medical imaging applications, which will close the gap between medical imaging and personalized medicine [11]. However, several challenges are also associated with radiomic analysis and interpretation. First of all, the same feature may yield different values under different image acquisition or pre-processing methods, leading to variability in feature stability. Secondly, a radiomic analysis typically produces thousands of features, with redundancy among them. This issue requires a feature selection or dimensionality reduction step, for which several approaches have been taken, ranging from selection of features based on low interobserver variability, low variance based on ROI permutations, or a data-driven approach using feature cross-correlation [12]. On the other hand, the relative merits of these approaches and their impact on model performance have not been well studied, and the results can vary depending on the chosen methods and parameter settings used to select the features. In addition, during the modelling stage, the diversity of model parameters and their adjustments often rely heavily on the engineer’s expertise, further increasing the uncertainty and subjectivity of the modelling process. These factors collectively present significant challenges for standardizing radiomics-based machine learning methods in practical clinical applications, despite the ongoing efforts to standardize radiomics methods, such as the Image Biomarker Standardization Initiative (IBSI) that has provided valuable research [13], complemented by studies exploring the impact of preprocessing methods on carotid CTA images [14] and investigating the importance of voxel and intensity quantification in CT images within oncology [15]. Finally, most existing studies primarily focus on patient or lesion images, with limited research utilizing healthy populations as a reference.

For these reasons, in this study, we aim to develop an interpretable and reproducible quantitative CCTA analysis, based on radiomics and machine learning using the cohort from the SCOT-HEART trial, selecting a stable subset of coronary radiomic features based on their stability in people without CAD. We compare the performance of using only stable features vs all of them in classification tasks at a patient level (examining calcified plaque burden) and at a segmental level (examining stenoses). Finally, we conduct Shapley analysis to measure key features’ marginal contribution in six ML models and to enhance the transparency of the models’ decision-making process.

## Methods

### Data processing and study design

This is a post hoc analysis of the multicentre randomized controlled SCOT-HEART trial, with approval from the regional ethics committee, and all participants provided their written informed consent. The trial targeted patients exhibiting symptoms indicative of suspected angina caused by coronary artery disease. The primary findings of the SCOT-HEART trial have been documented in prior publications [16–20].

### Computed tomography acquisition and analysis

All study participants in the current analysis underwent coronary artery calcium scoring and CCTA using a 320-multidetector (Aquilion ONE, Toshiba Medical Systems, Tochigi, Japan) and two 64-detector row scanners (Brilliance 64, Philips Healthcare, Netherlands; Biograph mCT, Siemens, Germany). Participants were scanned following the administration of GTN with a beta-blocker administered as required when the heart rate was > 60 beats per minute. Based on heart rate and rhythm, prospective or retrospective gating was used. CCTA was performed following an intravenous injection of 50 to 70 mL of iodine-based contrast medium at a flow rate of 5.5 to 6.5 mL/s. All participants in the intervention arm of the SCOT-HEART trial had complete CCTA imaging data, and thus, no missingness occurred for image-based analysis. For clinical variables obtained from participants’ electronic health records (EHRs), sourced through the electronic Data Research and Innovation Service (eDRIS) of NHS Scotland, continuous variables such as age and BMI with missing values were imputed using the mean. For categorical variables or parameters with a small number of missing entries, we excluded those entries from the analysis.

The coronary calcium score was calculated from the non-contrast-gated cardiac CT scan, using the Agatston technique [21]. The participants were categorized based on their coronary calcium scores into four groups: 0–10, 11–100, 101–399, and above 400 AU. The CCTA scans were analysed for the presence of stenosis at a segmental level. Segments were categorized as follows: no stenosis (stenosis < 10%), mild stenosis (stenosis of 10%–49%), moderate stenosis (stenosis: 50%–70%), and severe stenosis (stenosis of > 70%). For the current analysis, we focussed on the five main coronary segments which have the greatest prognostic importance: left main artery (LMA), left circumflex artery (LCX), left anterior descending artery (LAD), proximal right coronary artery (pRCA), and mid-right coronary artery (mRCA).

### Segmentation

To delineate the region of interest (ROI) around the coronary lumen, we engaged a semiautomated vessel segmentation tool, enhanced with the nnU-Net neural network algorithm for precise vessel delineation [22, 23]. An experienced radiologist (J. R. W. M., > 10-year experience in CCTA analysis) further refined these results through manual adjustments, ensuring the segmentation’s accuracy. After extracting the outer contour of the coronary lumen, we defined the periluminal region as a ring-shaped volume extending radially outward by two pixels. This region corresponds to the immediate perivascular space surrounding the coronary artery wall and is used to capture tissue characteristics adjacent to the lumen. This was selected to capture the immediate coronary wall, plaque, and adjacent fat without adding the incumbent variability inherent to plaque segmentation/contouring. Within this specified ROI, we extracted a total of 1834 radiomic features utilizing the PyRadiomics library [24] (version 3.1.0) including 7 categories (see Appendix file: Fig. S1). For the patient level coronary artery calcification evaluation, we treated the five major coronary segments (LMA, LCX, LAD, pRCA, and mRCA) as a whole ROI for feature extraction. For stenosis evaluation, which was performed at a segmental level, we extracted features separately from each ROI of the five major coronary segments (LMA, LCX, LAD, pRCA, and mRCA). The Hounsfield unit range of all CCTA images was resampled, limited to values from − 170 to 200 HU, as this covers both fat and plaque but avoids contamination from the adjacent lumen and focuses bin widths on the most relevant features of interest. All analyses were implemented using PyTorch and trained on an NVIDIA RTX 3080 GPU and Intel Core i9-12,900 CPU.

### Stable feature test

To select “stable” radiomic features that were not overly impacted by noise, reconstruction techniques, or ROI size, we examined these variables in 221 individuals (*CACS* = 0, coronary stenosis < 10%, without CAD). We compared the variation of these features across 21 different imaging settings. The CCTA images underwent resampling, employing a variety of isotropic and anisotropic voxel sizes. This selection encompasses standard sizes frequently [15], allowing us to encompass examples of both up-sampling and downsampling in our analysis (Appendix file: Fig. S2). The 21 imaging settings are as follows:

1. Original, i.e. no resampling.
2. Isotropic choices: (0.50, 0.50, 0.50) mm, (0.75, 0.75, 0.75) mm, (1.0, 1.0, 1.0) mm, (1.25, 1.25, 1.25) mm, and (1.5, 1.5, 1.5) mm.
3. For each resampling, four different interpolation methods were compared: linear, B-spline (spline), Welch windowed sinc interpolation, and nearest-neighbour interpolation.

These stable radiomic features formed the radiomic feature pre-selection approach, to be compared against all radiomic features without preselection. A feature was considered stable if the average variance between these resampling and interpolation techniques among the 221 individual subjects was less than 25% (*Δr* < 0.25) and highly stable if the variance was less than 10% (*Δr* < 0.1).

### Feature selection

The data-driven dimension reduction approach was performed using Pearson correlation analysis and LASSO regression. After performing Pearson correlation analysis on the features, for those with a Pearson correlation coefficient greater than 0.9, only one feature will be retained. The LASSO-CV model combines the least absolute shrinkage and selection operator (LASSO) technique with cross-validation (CV) methods to enhance model reliability and efficiency.

### Comparison of approaches

To compare the effect of the different data processing approaches, five models were used: (1) all radiomic features without feature selection, (2) stable radiomic features without data-driven feature reduction, (3) all radiomics features with data-driven feature selection, (4) stable radiomics features further augmented by data-driven feature selection, and (5) clinical factors in combination with model 4.

### Building of machine learning models

We employed six diverse algorithms to develop and evaluate models for assessing coronary calcification and segment-level stenosis severity based on CTA imaging. These algorithms encompass support vector machine (SVM), Gradient Boosting Classifier (GBC), Light Gradient Boosting Machine (LightGBM), K-nearest neighbours (KNN), Naive Bayes, and multilayer perceptron (MLP). SVM is employed to identify an optimal hyperplane that effectively differentiates between data point categories. GBC iteratively enhances weak classifiers, adjusting sample weights to prioritize previously misclassified samples. KNN assigns new samples to the most frequent category among its K-nearest neighbours. MLP (multilayer perceptron) is a type of neural network that learns complex patterns by passing data through multiple layers of interconnected neurons with non-linear activation functions. LightGBM is a gradient-boosting framework that builds decision trees sequentially, using a leaf-wise growth strategy to improve efficiency and accuracy, particularly on large datasets, and Naïve Bayes, a rapid and efficient probabilistic classifier, assumes independence between features and models their probabilities with a Gaussian distribution. The use of multiple machine learning models is not only aimed at accurately assessing coronary calcification and branch-level stenosis but also at validating whether the periluminal coronary CCTA features exhibit strong generalization performance across different machine learning models.

To reduce overfitting and to improve model generalisability, we employed fivefold cross-validation for model evaluation. In each round of cross-validation, a random selection of four-fifths of the data were used for training, with the remainder serving as the validation set, and 20% of the training data were randomly selected as a test set to assess model performance. ROC curves and AUC values are selected to quantify the discriminative capabilities and predictive accuracies of the models.

### Shapley additive explanations analysis

SHAP (SHapley Additive exPlanations) by Lundberg and Lee [25] is a method to explain individual predictions. The SHAP method interprets the prediction for instance x by calculating the contribution of each feature to a machine learning model. SHAP explanations are derived from cooperative game theory, computing Shapley values which distribute the output value fairly among the features. In essence, SHAP attributes the output value to each feature’s Shapley value, quantifying each feature’s impact on the final model output. For example, in medical statistics, the input could be a single feature value such as EHR data or a set of feature values. In this study, to explain CCTA images, features were extracted from coronary CT scans, and the SHAP method was employed to assign prediction weights across eight different machine learning models.

1 $$g\left({z}^{\prime}\right)={\phi }_{0}+\sum\nolimits_{j=1}^{M} {\phi }_{j}{z}_{j}^{\prime}$$

In this framework, $g\left({z}^{\prime}\right)$ represents the explanation model, *M* is the number of input features, and *z* indicates the presence (1 or 0) of corresponding features. Here, the presence pertains to contexts such as images and textual data (for instance, in text, after one-hot encoding a word, not all words appear in every sentence); ${\phi }_{j}$ denotes the attribution value (Shapley value) for each* j* feature, and ${\phi }_{0}$ is a constant. Since tree models use structured data as input, all features are present for a sample ${x}^{\prime}$; thus, the formula can be written as follows:

2 $$g\left({x}^{\prime}\right)={\phi }_{0}+\sum\nolimits_{j=1}^{M}\ {\phi }_{j}$$

We employed Cox regression analysis to investigate the association between radiomics features, clinical risk factors, and the clinical endpoint of myocardial infarction. To assess the independent effect of each covariate on survival time while controlling for the influence of other variables, we first performed univariate survival analysis. Based on the identification of potential risk factors, we subsequently conducted multivariate Cox regression to evaluate the joint effects of multiple covariates on the clinical outcome. In addition, bootstrap was used to assess the stability and variability of model performance metrics, such as the time-dependent concordance index.

## Results

### Baseline characteristics of the study populations

The study cohort which included 909 participants was divided into two parts.

- Six-hundred eighty-one participants (mean age = 58.27 ± 9.11, male sex = 392/57.56%) were used for model training and internal validation. Of these, 193 had no stenosis, 175 had mild stenosis, 124 had moderate stenosis, and 189 had severe stenosis. Those with more severe stenosis were more likely to be male and have hypertension and a history of coronary artery disease. There were 14 participants with the endpoint (fatal or nonfatal myocardial infarction). All the detailed characteristics are shown in Table 1 and Appendix file: Fig. S1.
- A total of 228 CCTA cases acquired on two 64-detector row scanners were incorporated for external validation (shown in Appendix file: Table S4).

**Table 1:** Characters baseline of participants' population

Characteristic	Participants *N* = 681	No significant CAD *N* = 193	Mild CAD *N* = 175	Moderate CAD *N* = 124	Severe CAD *N* = 189	*p*-value
Age	58.27 ± 9.11	53.04 ± 9.71	60.67 ± 7.93	58.83 ± 8.64	61.20 ± 7.36	< 0.001
Gender						< 0.001
Female — *n* (%)	289 (42.44)	118 (61.14)	79 (45.14)	46 (37.10)	46 (24.34)	
Male — *n* (%)	392 (57.56)	75 (38.86)	96 (54.86)	78 (62.90)	143 (75.66)	
Total cholesterol (mmol/L)	4.96 ± 1.96	4.88 ± 1.95	4.84 ± 2.14	5.14 ± 1.72	5.0 ± 1.99	0.451
HDL cholesterol	0.87 ± 0.70	0.87 ± 0.76	0.83 ± 0.70	0.98 ± 0.65	0.84 ± 0.65	0.255
Body mass index (kg/m^2^)	29.23 ± 5.01	29.09 ± 5.72	29.06 ± 4.84	29.61 ± 5.28	29.25 ± 4.18	0.779
Hypertension — *n* (%)	234 (34.36)	49 (25.39)	57 (32.57)	46 (37.10)	82 (43.39)	< 0.001
CHD prior history — *n* (%)	62 (9.10)	4 (2.07)	10 (5.71)	18 (14.52)	30 (15.87)	< 0.001
Smoking history — *n* (%)	372 (54.63)	95 (49.22)	89 (50.86)	84 (67.74)	104 (55.03)	0.0062
CHD family history — *n* (%)	275 (40.38)	75 (38.86)	73 (41.71)	46 (37.10)	81 (42.86)	0.875
Diabetes — *n* (%)	83 (12.19)	20 (10.36)	22 (12.57)	16 (12.90)	25 (13.23)	0.832
Cerebrovascular disease — *n* (%)	32 (4.70)	5 (2.59)	9 (5.14)	11 (8.87)	7 (3.7)	0.083
CAC score (AU)	286 ± 706	2 ± 13	70 ± 78	343 ± 696	737 ± 1070	< 0.001

Mean (standard deviation); number (percent); *CAC *coronary artery calcium, *CHD* coronary heart disease, *BMI* body mass index

### Stable features in participants without CAD

**Fig. 1:**
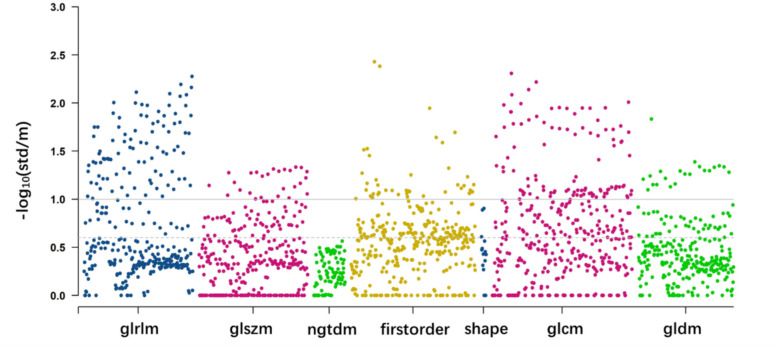
Manhattan plot of the distribution of 1834 radiomics feature value stability in the population without CAD. The horizontal axis represents the seven categories of radiomics features. Each point represents a specific radiomics feature. The dotted line is the threshold of stable features; the solid line represents the threshold of highly stable features

**Fig. 2:**
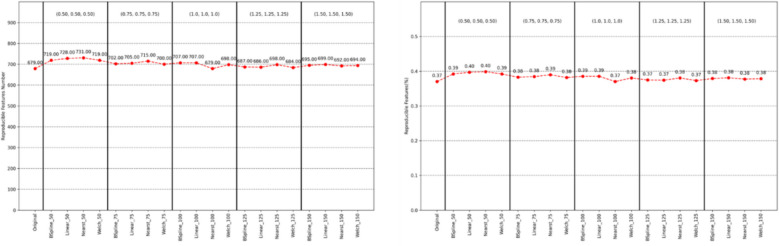
Stability statistics and percentage plot of features in the population without CAD

We analysed the distribution of CTA radiomic features in 221 individuals (*CACS* = 0, stenosis < 10%, without CAD) across 21 different imaging configurations to assess the stability of these features. This evaluation aimed to identify features that could enhance the reproducibility of subsequent machine learning models. Additionally, we investigated the dependence of CTA imaging features on voxel size and interpolation methods. Of 1834 features, 549 remained stable across all 21 imaging configurations (*Δr* < 0.25; Fig. 1). Approximately, 66% to 77% of the features are stable under a specific imaging configuration among the 21 imaging configurations, with around 20% to 33% being highly stable (Fig. 2 and Appendix file: Figs. S2 and S3).

### Clinical and biological relevance of stable radiomic features

To assess the clinical value of the selected stable radiomic features, we performed a Cox proportional hazards regression analysis to evaluate their associations with the occurrence of myocardial infarction (MI) over a 5-year follow-up period. The results are summarized as hazard ratios with 95% confidence intervals and visualized using forest plots (Appendix file: Fig. S6; Appendix file: Table S2). Several features demonstrated significant prognostic value, indicating their potential to stratify patients at increased risk of MI.

In parallel, to support the biological plausibility of these features, we examined their correlations with coronary artery calcium score (CACS), a well-established marker of atherosclerotic plaque burden. As shown in Appendix file: Supplemental Table 3 and Supplemental Fig. 7, multiple features exhibited strong and statistically significant correlations with CACS. For instance, squareroot_ngtdm_Strength (Spearman *ρ* = 0.849) and gradient_ngtdm_Strength (*ρ* = 0.803) showed robust positive associations, suggesting that increased myocardial textural strength is linked to higher calcification burden. In contrast, log_sigma_2_0_mm_3D_glcm_InverseVariance (*ρ* = − 0.722) was negatively correlated with CACS, potentially reflecting reduced local homogeneity in patients with more advanced plaque.

Together, these analyses provide converging evidence that the identified stable features are not only statistically robust but also clinically prognostic and biologically interpretable, supporting their potential utility in cardiovascular risk assessment and patient stratification.

### Comparison of feature selection pipelines for predicting coronary artery calcification score category

The average calcification score among all participants was 286 ± 706 AU. A total of 201 individuals had calcification scores in the range of 0–10, 165 had scores between 11 and 100, 125 had scores ranging from 101 to 399, and 109 had scores exceeding 400. The five feature selection pipelines were compared using six classical machine learning algorithms (Table 2).

**Table 2:** Performance of coronary calcification assessment models

	All features based		Unstable features based		Stable features based		Stable + clinical features based	
Machine learning models	Train (95% CI)	Test (95% CI)	Train (95% CI)	Test (95% CI)	Train (95% CI)	Test (95% CI)	Train (95% CI)	Test (95% CI)
SVM	0.885 (0.855, 0.909)	0.842 (0.775, 0.897)	0.754 (0.715–0.793)	0.733 (0.654–0.802)	0.883 (0.853, 0.907)	0.800 (0.72, 0.86)	0.908 (0.881, 0.930)	0.808 (0.735, 0.866)
KNN	0.827 (0.794, 0.857)	0.750 (0.671, 0.815)	0.723 (0.683–0.763)	0.708 (0.627–0.779)	0.800 (0.764, 0.831)	0.742 (0.663, 0.809)	0.812 (0.778, 0.843)	0.708 (0.624, 0.776)
LightGBM	0.931 (0.906, 0.949)	0.825 (0.751, 0.878)	0.848 (0.816–0.880)	0.735 (0.656–0.794)	0.908 (0.881, 0.930)	0.752 (0.671, 0.815)	0.919 (0.893, 0.939)	0.752 (0.671, 0.815)
Gradient boosting	0.896 (0.867, 0.918)	0.750 (0.671, 0.815)	0.817 (0.782–0.852)	0.703 (0.612–0.775)	0.892 (0.863, 0.915)	0.743 (0.663, 0.809)	0.885 (0.855, 0.909)	0.733 (0.655, 0.802)
MLP	0.883 (0.853, 0.907)	0.833 (0.759, 0.885)	0.783(0.746–0.819)	0.750 (0.673–0.807)	0.871 (0.841, 0.897)	0.783 (0.703, 0.841)	0.869 (0.839, 0.895)	0.800 (0.727, 0.860)
Naïve Bayes	0.800 (0.764, 0.831)	0.785 (0.711, 0.847)	0.723 (0.683–0.763)	0.708 (0.627–0.789)	0.783 (0.747, 0.816)	0.767 (0.687, 0.828)	0.796 (0.760, 0.828)	0.783 (0.703, 0.841)

Of the five models, the model based on all radiomic features with no feature reduction exhibited the poorest performance. It showed signs of overfitting, with a training accuracy of 1, and a marked performance drop within the testing cohort, with the poorest accuracy of the five approaches across all six machine learning models. The model using stable radiomic features without further data-driven feature reduction also showed model overfitting with training performances of 0.98–1 but a marked drop-off in the testing cohorts albeit marginally less than using all the radiomic features.

Of the two approaches using data-driven feature selection (Pearson correlation and LASSO), the pipeline which started with all radiomic features exhibited higher accuracy in training and testing as well as a smaller drop between these two groups than the pipeline using the stable features. In the testing set, this difference between the two pipelines ranged from 0.007 to 0.073 across the six machine learning models. The magnitude of this benefit was comparable to the difference between the best and poorest performing model (0.092).

**Fig. 3:**
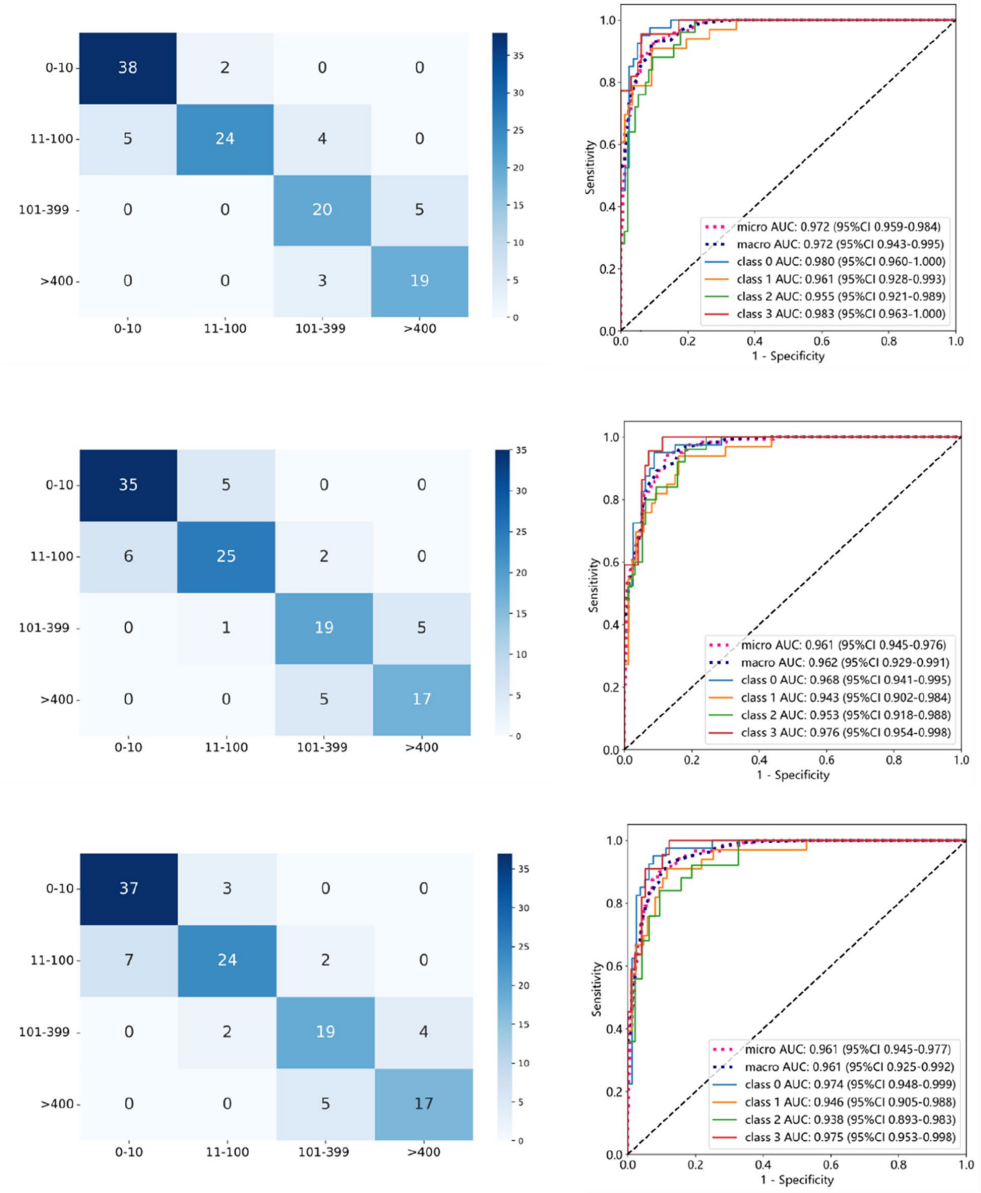
Performance of the coronary calcification quantification in machine learning models. Top left: Heatmap of the all-feature-based model prediction results in the test set; top right: AUC plot of the all-feature-based model in the test set. Middle: The stable feature-based machine learning model performance. Bottom: Stable radiomics features and clinical factors combined machine learning model performance. The heatmap summarizes the classification results for the four calcium score categories. Each cell shows how often the model’s predictions matched the actual diagnosis, helping identify which types are most accurately recognized and where misclassifications occur. The ROC (receiver operating characteristic) curves illustrate the model’s ability to distinguish each category. The area under the curve (AUC) provides a measure of this performance: AUC values range from 0.5 (no better than chance) to 1.0 (perfect classification); AUC values above 0.9 are considered excellent, indicating that the model can reliably differentiate between four types; macro-AUC represents the average performance across all classes, treating each class equally; micro-AUC reflects the overall performance by considering all instances together, giving more weight to common classes. Together, these metrics offer a comprehensive view of the model’s diagnostic accuracy

Among the six algorithms, SVM, MLP, and LightGBM achieved prediction accuracies exceeding 80%. In contrast, KNN, Naïve Bayes, and gradient boosting attained accuracies between 75 and 80%. The SVM algorithm outperformed the others, achieving a four-class classification accuracy of 84.2% for predicting CAC categories. The AUCs for the SVM model across the classes were as follows: class_0 (*CACS*: 0–10): 0.980; class_1 (*CACS*: 11–100): 0.961; class_2 (*CACS*: 101–399): 0.955; and class_3 (CACS: ≥ 400): 0.983. The micro-average AUC was 0.972, and the macro-average AUC was also 0.972 (Fig. 3 and Appendix file: Table S5).

When comparing the models based on stable imaging features with those that combined clinical risk factors and stable imaging features, the models incorporating clinical factors showed only a slight improvement in predicting the severity of CAC and still did not bring model performance up to the levels of models starting with all the available radiomic features. For instance, with the SVM algorithm, the combined model increased accuracy by 0.025 in the training set and by just 0.008 in the test set compared to the model based solely on stable imaging features. The best model (SVM) maintained consistent performance across these datasets (accuracy: 0.811, *AUC*: 0.939) reported in Appendix file: Fig. S8.

### Comparison of feature selection for predicting coronary artery stenosis

**Fig. 4:**
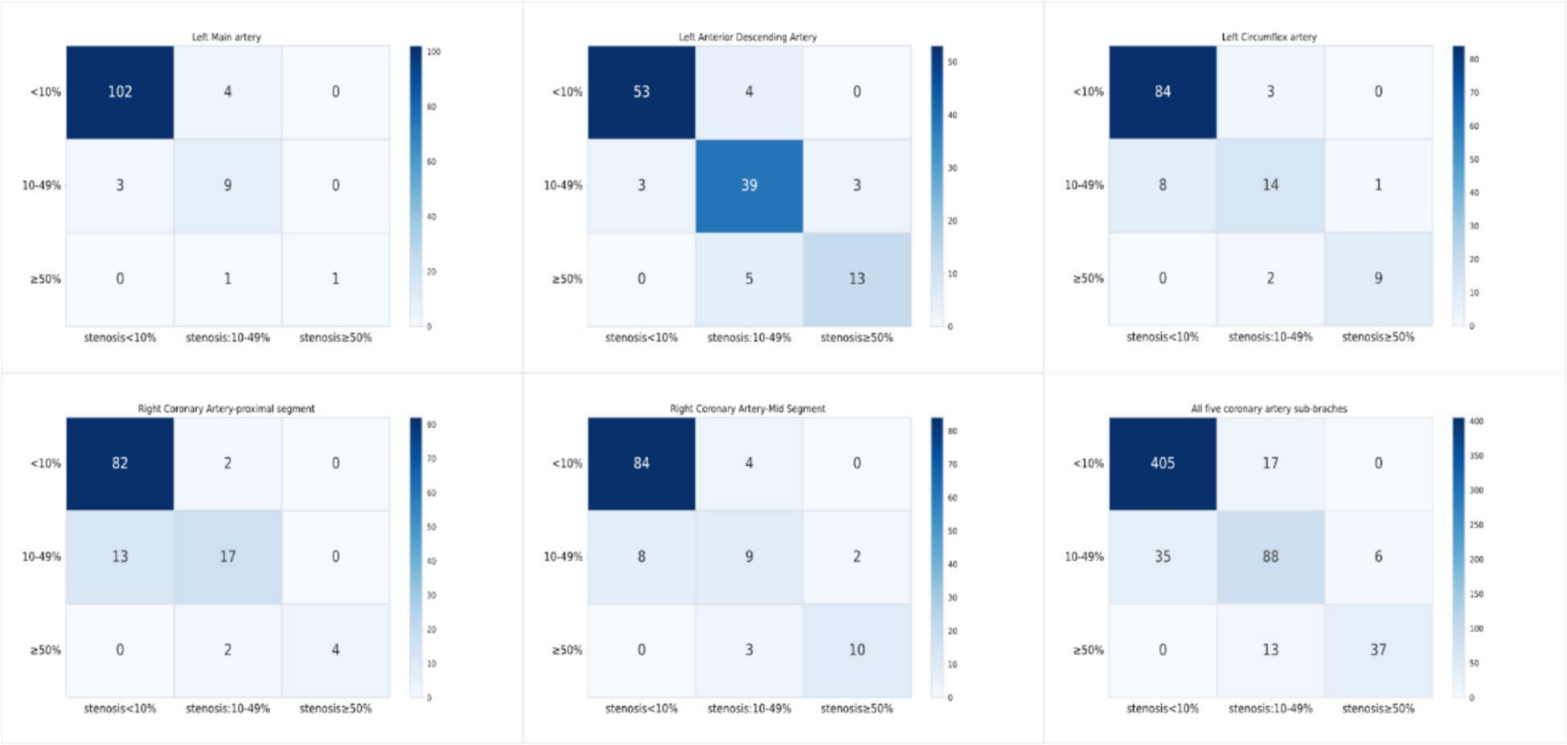
Performance of coronary segment-level stenosis assessment machine learning model. The six heatmaps present model assessment performance for five major coronary segments’ stenosis (LMA, LCX, LAD, pRCA, and mRCA). The bottom right figure shows the whole five coronary segments’ prediction results

In this cohort study, we examined a total of 3083 segments of coronary arteries. Among these, 2038 artery segments had a stenosis degree of less than 10%, 760 artery segments had stenosis between 10 and 49%, and 285 artery segments exhibited stenosis greater than 50%. In our study, the prediction model demonstrated high accuracy in assessing stenosis in the five major coronary branches. In the test set, the model achieved a prediction accuracy of over 80% in all territories (Table 3 and Fig. 4).

**Table 3:** Performance for major coronary segments’ stenosis classification in SVM model

Segments	Total	Stenosis < 0%	Stenosis 10–49%	Stenosis ≥ 50%	Train accuracy (95% *CI*)	Test accuracy (95% *CI*)
All segments	3083	2038	760	285	87.3% (85.9–88.6%)	86.7% (83.8–89.2%)
pRCA	614	446	132	36	86.7% (83.5–89.5%)	84.4% (77.1–89.9%)
mRCA	658	445	130	83	86.9% (83.7–89.5%)	84.2% (76.9–89.4%)
LMA	518	415	89	14	93.9% (91.2–95.9%)	92.7% (85.6–96.1%)
LCX	643	450	149	44	92.9% (90.5–94.9%)	89.2% (82.6–93.4%)
LAD	650	282	260	108	85.8% (82.5–88.5%)	84.5% (77.4–89.8%)

*pRCA* right coronary artery-proximal segment, *mRCA* right coronary artery-mid segment, *LMA* left coronary artery, *LCX* left circumflex artery, *LAD* left anterior descending artery

**Fig. 5:**
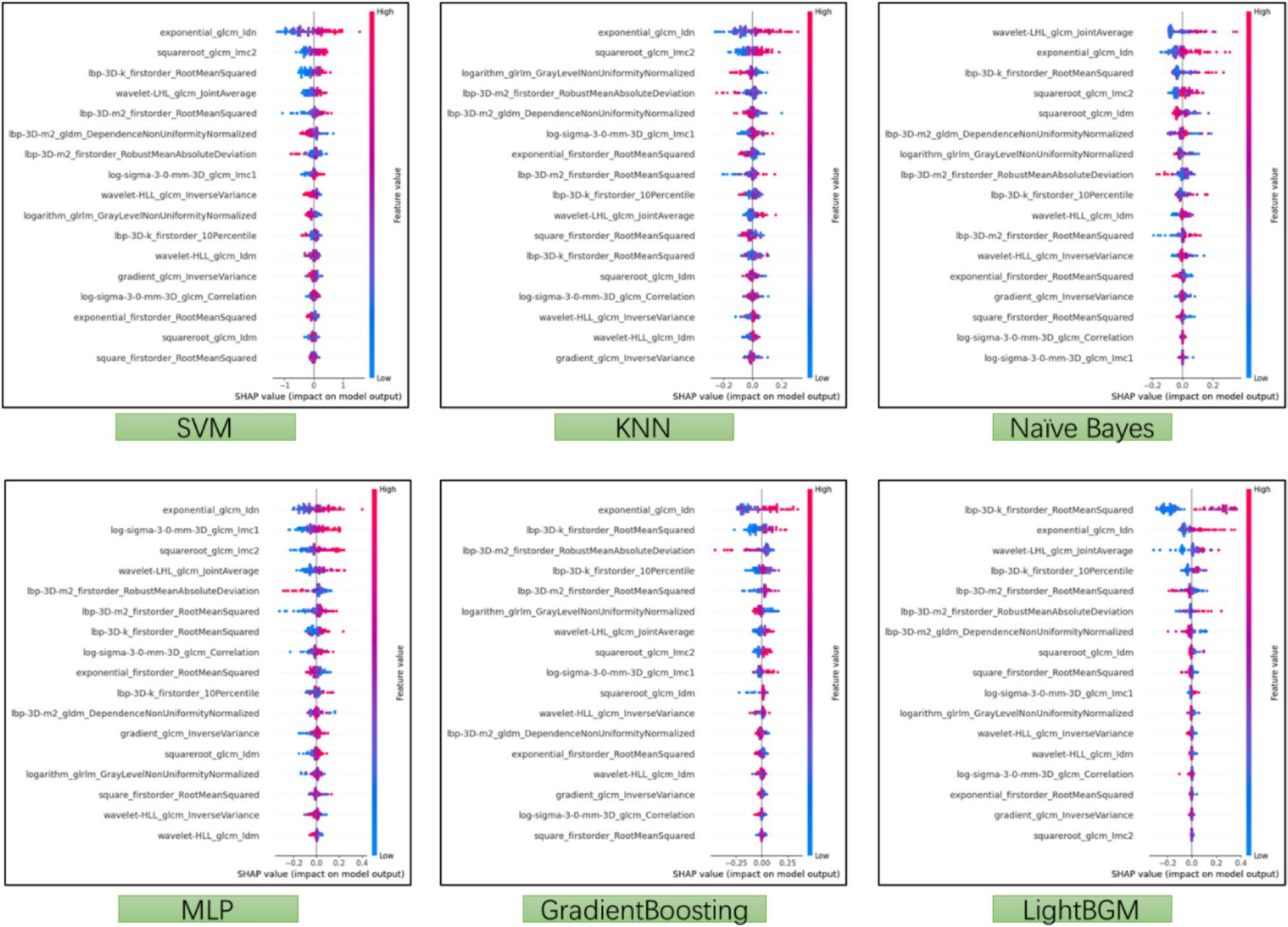
Radiomics feature importance Shapley summary chart. Each dot represents a specific participant. The dot plot illustrates the direction of contribution for each feature, with red indicating higher values and blue indicating lower values for each variable

**Fig. 6:**
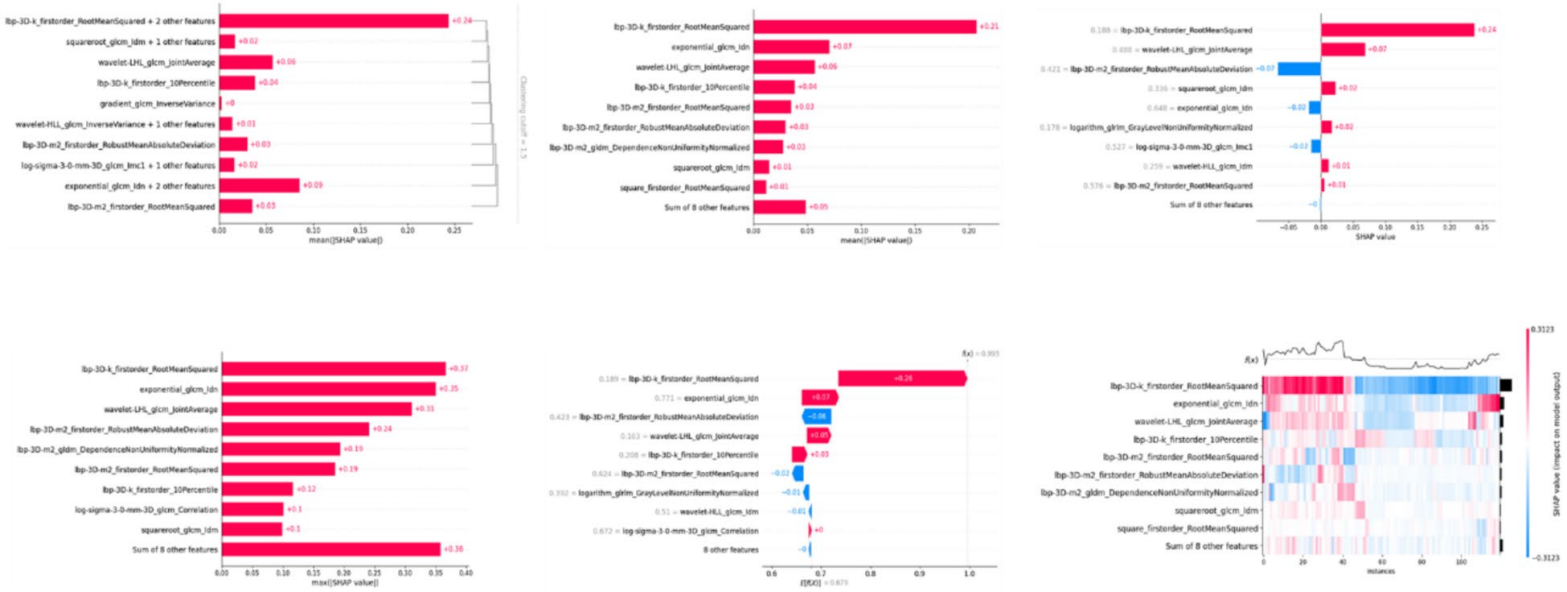
Local model explanation by the Shapley method

When comparing the outcomes between the model built on all radiomic features and the model based on stable radiomic features, the performances in evaluating stenosis across the five main coronary segments were very similar. In both the test and training sets, the difference in prediction accuracy was less than 0.03. This consistency in performance is observed across all six machine learning algorithms used in the study (Appendix file: Table S1). Further, Shapley analysis reveals that the contribution of each imaging feature to the model’s decision-making process varies across different machine learning models (Figs. 5 and 6, Appendix file: Fig. S4 and Table S5).

## Discussion

In this study, we systematically evaluated the stability of coronary and periluminal radiomic features extracted from CCTA across 21 different image preprocessing settings in individuals without CAD. We further examined how feature stability influences ML model performance for coronary calcification and stenosis assessment. Importantly, we found that preselecting features based on their stability did not lead to major performance degradation in either the training or testing phases, regardless of the algorithm used. These results suggest that prioritizing stability during feature selection can enhance model reproducibility and generalizability without compromising diagnostic accuracy.

To the best of our knowledge, this is the first study to comprehensively investigate the distribution and stability of periluminal coronary features derived from CCTA in a CAD-free population. From 1834 extracted features, 549 were identified as stable across preprocessing configurations. These stable features provide a reference framework for future CCTA-based quantitative imaging studies. Previous research on feature reproducibility has largely focused on oncologic [14] or carotid imaging [15], with limited exploration in cardiovascular applications. Our study delves into the properties of various features of CCTA in a CAD-free cohort, selects stable features, and applies multiple machine learning methods for validation in patients. This investigation may contribute to the standardization of radiomics in cardiovascular imaging research.

Radiomic ML models built using these stable features demonstrated robust performance in quantifying coronary calcification, achieving accuracies exceeding 80% in four-class calcium scoring. This performance is comparable to data-driven models using all features, indicating that carefully selected stable features retain the discriminative power necessary for reliable calcium quantification. Importantly, several of these features showed strong correlations with CACS, a well-established marker of atherosclerotic burden. These findings highlight the potential role of stable radiomic features in improving the consistency of automated coronary calcium assessment and supporting long-term cardiovascular risk evaluation. Previous studies have developed deep-learning-based models for automated calcium scoring [26–28], achieving high accuracy but often limited by small sample sizes and the opaque, “black-box” nature of deep networks. By contrast, our approach leverages a large, randomized, controlled dataset encompassing multiple scanners and imaging conditions. This diversity strengthens the external validity of our results.

In addition to calcium scoring, we extended our analysis to coronary stenosis classification at the segmental level. Using more than 3000 coronary segments from SCOT-HEART participants, we developed interpretable ML models that achieved strong predictive accuracy for stenosis stratification across the major coronary segments (LMA, LCX, LAD, pRCA, and mRCA). Compared with prior ML studies, whether using invasive ICA or CT imaging [29], that primarily evaluated stenosis at the patient level, our automated branch-level evaluation model achieved good accuracy for segment-level stenosis classification.

To enhance model transparency and interpretability, we employed Shapley value analysis to quantify the marginal contribution of each imaging feature to model predictions. This framework revealed that the importance of individual features varied across six algorithms, reflecting distinct learning mechanisms inherent to each modelling approach. Such interpretability will be helpful for understanding how quantitative imaging features influence diagnostic predictions and for establishing trust in AI-assisted cardiovascular diagnostics. Shapley-based feature attribution has previously been applied in kidney injury [30] and hepatic inflammation [31] research, and our work extends this approach to CCTA.

Our findings suggest the importance of identifying features that are both statistically stable and clinically relevant. Radiomic features that remain consistent across different imaging processing settings may have improved potential to generalize across institutions, patient populations, and acquisition protocols. Models developed using such features could help reduce the risk of overfitting site-specific characteristics, thereby enhancing reproducibility and interpretability. From a translational perspective, reproducible and interpretable ML frameworks may assist in promoting more consistent quantitative CCTA assessment results in different situations.

This study has several limitations that should be acknowledged but also provides clear directions for future research. The overall sample size and scanner diversity remain limited. Future studies should therefore involve larger, multicentre cohorts with a wider range of imaging protocols and patient characteristics to further validate and generalize our findings. In addition, while a single, widely used feature reduction approach (Pearson correlation filtering combined with LASSO regression) was adopted for interpretability, alternative dimensionality reduction and feature selection strategies—such as mutual information filtering, recursive feature elimination, or tree-based embedded methods—will be explored to enhance model robustness. Future work will also include a prospective, head-to-head comparison between the AI model and expert readers to assess interpretability, inter-reader variability, and clinical consistency. Finally, further validation in more diverse and diseased populations, including patients with both stable and unstable coronary plaques, will be essential to ensure the stability and discriminative power of the proposed features across the full spectrum of CAD.

## Conclusions

We identified stable coronary and periluminal CCTA features from participants without CAD. The machine learning model developed using these stable features demonstrated good performance in both coronary calcification assessment and major coronary segments stenosis quantification, achieving comparable accuracy to purely data-driven models. These findings highlight the potential value of reproducible and interpretable models in advancing CCTA-based research and diagnosis of coronary artery disease.

## Supplementary Information

Supplementary Material 1: Supplemental Table 1. Performance of coronary segments stenosis assessment results crossing 6 machine learning methods. Supplemental Table 2. Associations Between Key Imaging Features and Myocardial Infarction Based on Univariable Cox Proportional Hazards Analysis. Supplemental Table 3. Spearman correlation between selected radiomic features and coronary artery calcium score (CACS). Supplemental Table 4. Characters baseline of external validation population. Supplemental Table 5. Prediction Performance of the ML Model Across CAC Score Categories. Supplemental Table 6. Prediction Performance of the ML Model Across Different Stenoses Severity. Supplemental Fig. 1. CONSORT flow diagram of participants within the current study. Supplemental Fig. 2. Combined violin and box plots of the distribution of radiomics feature value in individuals without CAD crossing different imaging processing configures. Supplemental Fig. 3. The feature value distribution variance plot in the CAD-free population. Supplemental Fig. 4. Shapley Additive explanations plot: the impact of stable radiomics features for diagnosing major coronary segments stenosis in the SVM, KNN, Naïve Bayes, MLP, Gradient Boosting, and Light BGM. Supplemental Fig. 5. Comparison of model accuracy across feature sets. Supplemental Fig. 6. Forest plot of the associations between key imaging features and myocardial infarction based on univariable Cox proportional hazards regression. Supplemental Fig. 7. Correlation between selected radiomic features and coronary artery calcium score (CACS). Supplemental Fig. 8. External Validation of Coronary Calcium Quantification Performance.

Supplementary Material 2. The list of stable features.

Supplementary Material 3. *P*-values for inter-group comparisons.

## Data Availability

This post hoc study is based on SCOT-HEART Trial, and no dataset was generated in current research.

## Abbreviations

AI: Artificial intelligence
AU: Agatston units
CACS: Coronary artery calcium score
CHD: Coronary heart disease
CCTA: Coronary computed tomography angiography
EHR: Electronic health record
ICA: Invasive coronary angiography
CT: Computed tomography
CV: Cross-validation
CVD: Cardiovascular disease
GBC: Gradient Boosting Classifier
GLCM: Grey-level co-occurrence matrix
GLDM: Grey-level dependence matrix
GLRLM: Grey-level run length matrix
GLSZM: Grey-level size zone matrix
KNN: K-nearest neighbours
LAD: Left anterior descending artery
LASSO: Least absolute shrinkage and selection operator
LCX: Left circumflex artery
LightGBM: Light Gradient Boosting Machine
LMA: Left main artery
MI: Myocardial infarction
MLP: Multilayer perceptron
mRCA: Mid-right coronary artery
NGTDM: Neighbouring grey tone difference matrix
pRCA: Proximal right coronary artery
ROI: Region of interest
SVM: Support vector machine
