# Supplementary material for: Interpretable and reproducible machine learning model for coronary calcification and segment-level stenoses stratification on computed tomography angiography
Source: BMC Med. 2025 Nov 26;23:657. doi: 10.1186/s12916-025-04478-0 (PMC12659364; doi:10.1186/s12916-025-04478-0)
Supplement: Supplementary file 1 — Supplementary Material 1: Supplemental Table 1. Performance of coronary segments stenosis assessment results crossing 6 machine learning methods. Supplemental Table 2. Associations Between Key Imaging Features and Myocardial Infarction Based on Univariable Cox Proportional Hazards Analysis. Supplemental Table 3. Spearman correlation between selected radiomic features and coronary artery calcium score (CACS). Supplemental Table 4. Characters baseline of external validation population. Supplemental Table 5. Prediction Performance of the ML Model Across CAC Score Categories. Supplemental Table 6. Prediction Performance of the ML Model Across Different Stenoses Severity. Supplemental Fig. 1. CONSORT flow diagram of participants within the current study. Supplemental Fig. 2. Combined violin and box plots of the distribution of radiomics feature value in individuals without CAD crossing different imaging processing configures. Supplemental Fig. 3. The feature value distribution variance plot in the CAD-free population. Supplemental Fig. 4. Shapley Additive explanations plot: the impact of stable radiomics features for diagnosing major coronary segments stenosis in the SVM, KNN, Naïve Bayes, MLP, Gradient Boosting, and Light BGM. Supplemental Fig. 5. Comparison of model accuracy across feature sets. Supplemental Fig. 6. Forest plot of the associations between key imaging features and myocardial infarction based on univariable Cox proportional hazards regression. Supplemental Fig. 7. Correlation between selected radiomic features and coronary artery calcium score (CACS). Supplemental Fig. 8. External Validation of Coronary Calcium Quantification Performance. [file 12916_2025_4478_MOESM1_ESM.docx]

**Supplemental Table 1.** Performance of coronary segments stenosis assessment results crossing 6 machine learning methods.

|  | pRCA | | | | mRCA | | | | LMA | | | | LCX | | | | LAD | | | |
| --- | --- | --- | --- | --- | --- | --- | --- | --- | --- | --- | --- | --- | --- | --- | --- | --- | --- | --- | --- | --- |
|  | All features | | Stable features | | All features | | Stable features | | All features | | Stable features | | All features | | Stable features | | All features | | Stable features | |
| machine learning models | Train | Test | Train | Test | Train | Test | Train | Test | Train | Test | Train | Test | Train | Test | Train | Test | Train | Test | Train | Test |
| SVM | 0.879 | 0.848 | 0.867 | 0.844 | 0.883 | 0.858 | 0.869 | 0.842 | 0.963 | 0.943 | 0.939 | 0.927 | 0.923 | 0.842 | 0.929 | 0.892 | 0.890 | 0.875 | 0.858 | 0.845 |
| KNN | 0.829 | 0.800 | 0.827 | 0.825 | 0.875 | 0.848 | 0.863 | 0.817 | 0.935 | 0.925 | 0.933 | 0.894 | 0.883 | 0.856 | 0.891 | 0.865 | 0.833 | 0.825 | 0.812 | 0.758 |
| LightGBM | 0.860 | 0.825 | 0.857 | 0.850 | 0.894 | 0.833 | 0.873 | 0.825 | 0.949 | 0.919 | 0.937 | 0.935 | 0.902 | 0.808 | 0..910 | 0.842 | 0.877 | 0.825 | 0.879 | 0.842 |
| Gradient Boosting | 0.865 | 0.825 | 0.840 | 0.825 | 0.900 | 0.858 | 0.887 | 0.842 | 0.972 | 0.919 | 0.959 | 0.927 | 0.916 | 0.833 | 0.887 | 0.858 | 0.879 | 0.833 | 0.875 | 0.808 |
| MLP | 0.842 | 0.823 | 0.833 | 0.819 | 0.881 | 0.833 | 0.869 | 0.842 | 0.949 | 0.919 | 0.943 | 0.919 | 0.916 | 0.833 | 0.883 | 0.877 | 0.869 | 0.842 | 0.844 | 0.842 |
| Naïve Bayes | 0.817 | 0.760 | 0.783 | 0.758 | 0.827 | 0.812 | 0.825 | 0.803 | 0.919 | 0.903 | 0.903 | 0.902 | 0.839 | 0.833 | 0.821 | 0.817 | 0.800 | 0.771 | 0.767 | 0.727 |

**Supplemental Table 2.** Associations Between Key Imaging Features and Myocardial Infarction Based on Univariable Cox Proportional Hazards Analysis.

**Supplemental Table 3.** Spearman correlation between selected radiomic features and coronary artery calcium score (CACS).

**
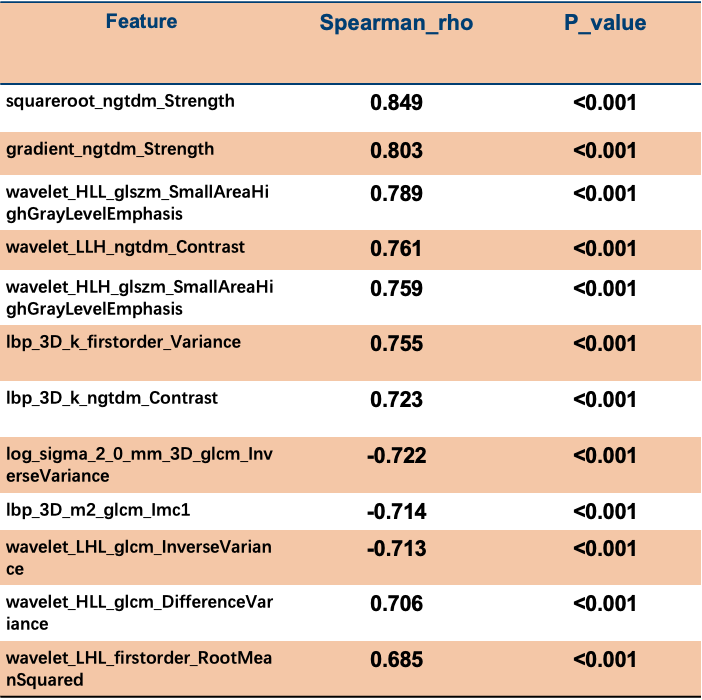
**

P-values were adjusted for multiple comparisons using the Bonferroni correction.

**Supplemental Table 4.** Characters baseline of external validation population.


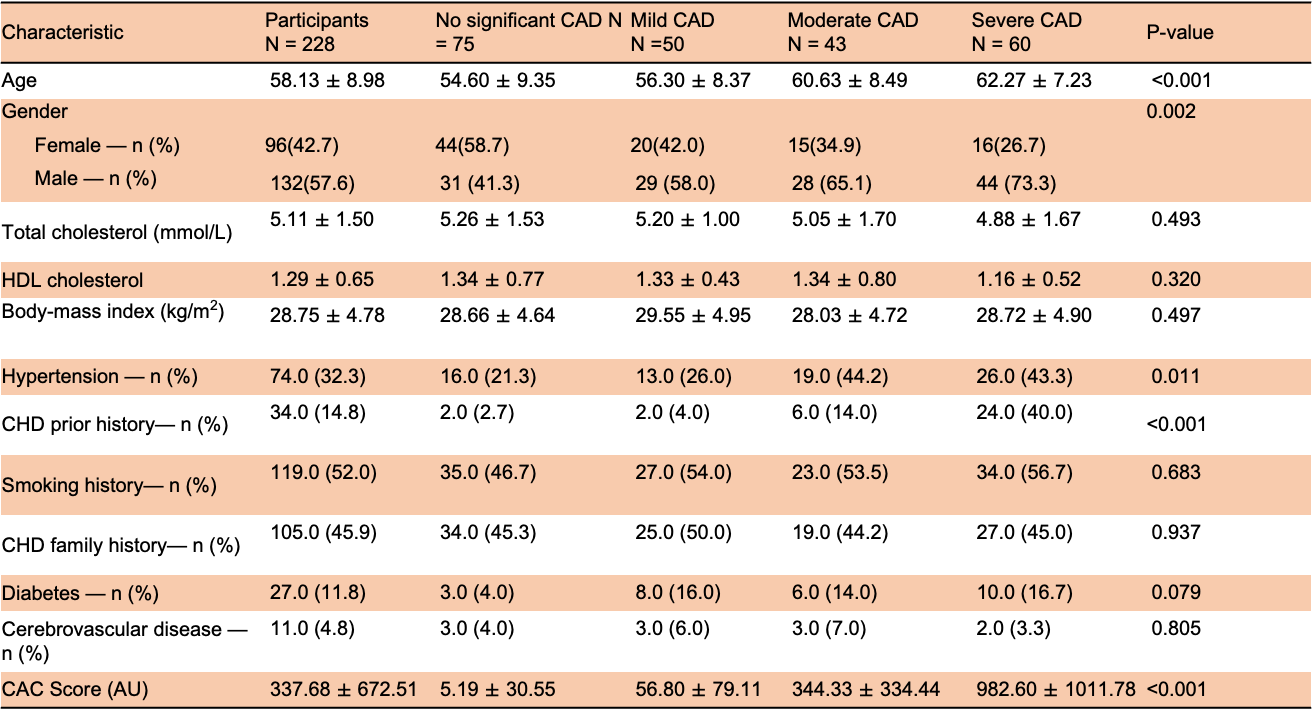


**Supplemental Table 5.** Prediction Performance of the ML Model Across CAC Score Categories


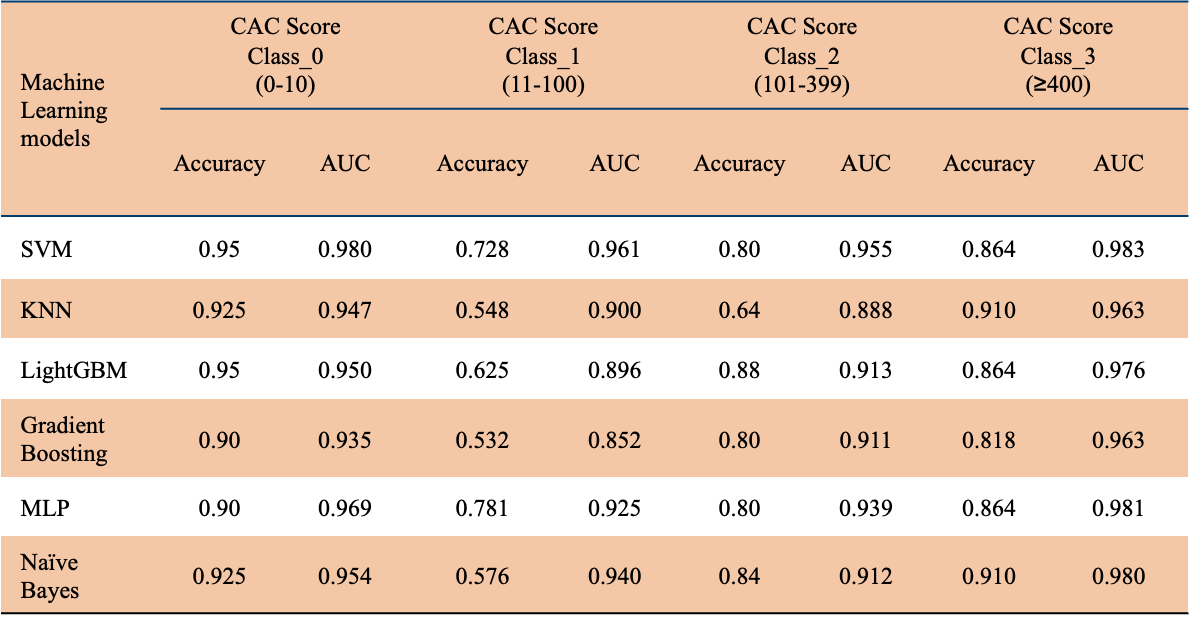


**Supplemental Table 6.** Prediction Performance of the ML Model Across Different Stenoses Severity.


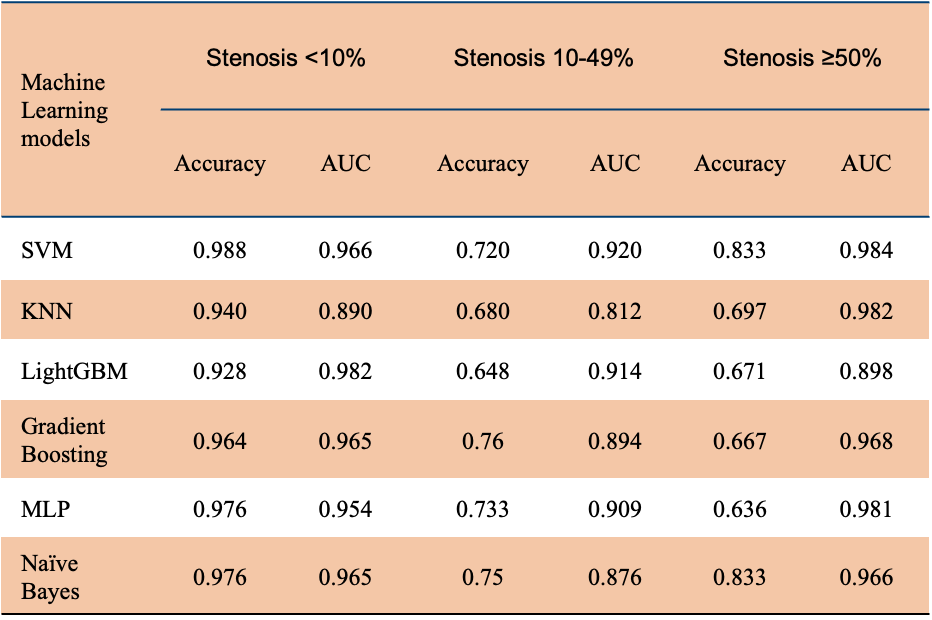


**Supplemental Figure 1:** CONSORT flow diagram of participants within the current study.


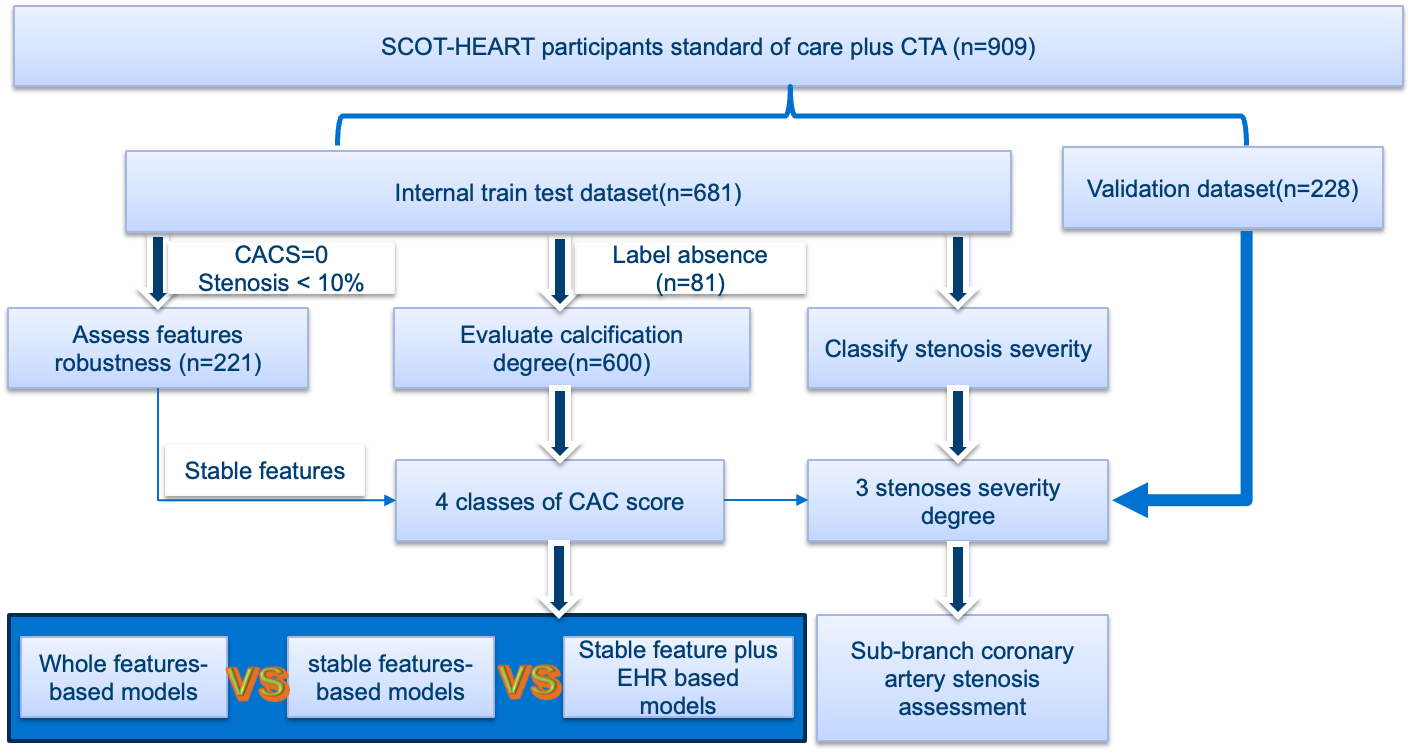


**Supplemental Figure 2:** Combined violin and box plots of the distribution of radiomics feature value in healthy individuals crossing different imaging processing configures.


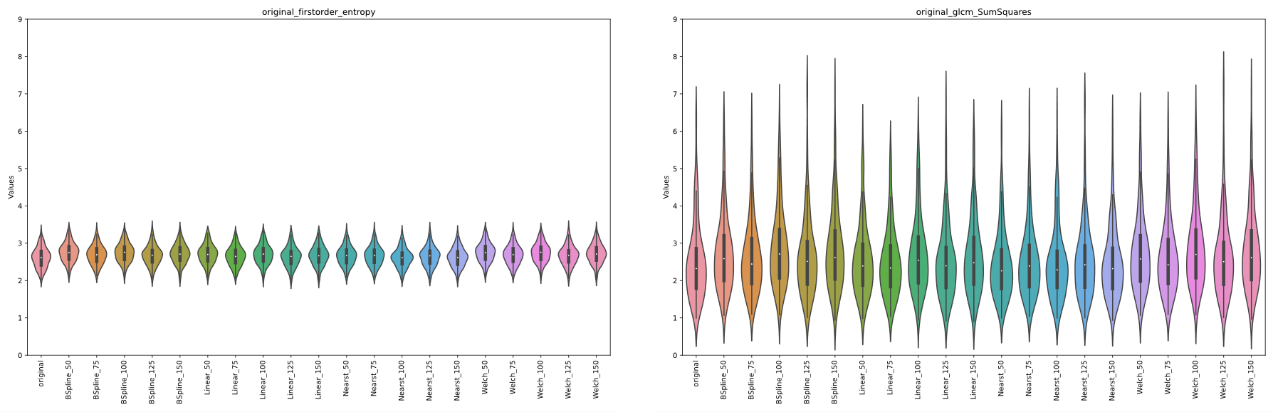


The horizontal axis represents the 21 imaging processing configures. The vertical axis represents the feature value. The left plot is original_firstorder_entropy; the right is original_glcm_sumsquares.

**Supplemental Figure 3:** The feature value distribution variance plot in the healthy population.


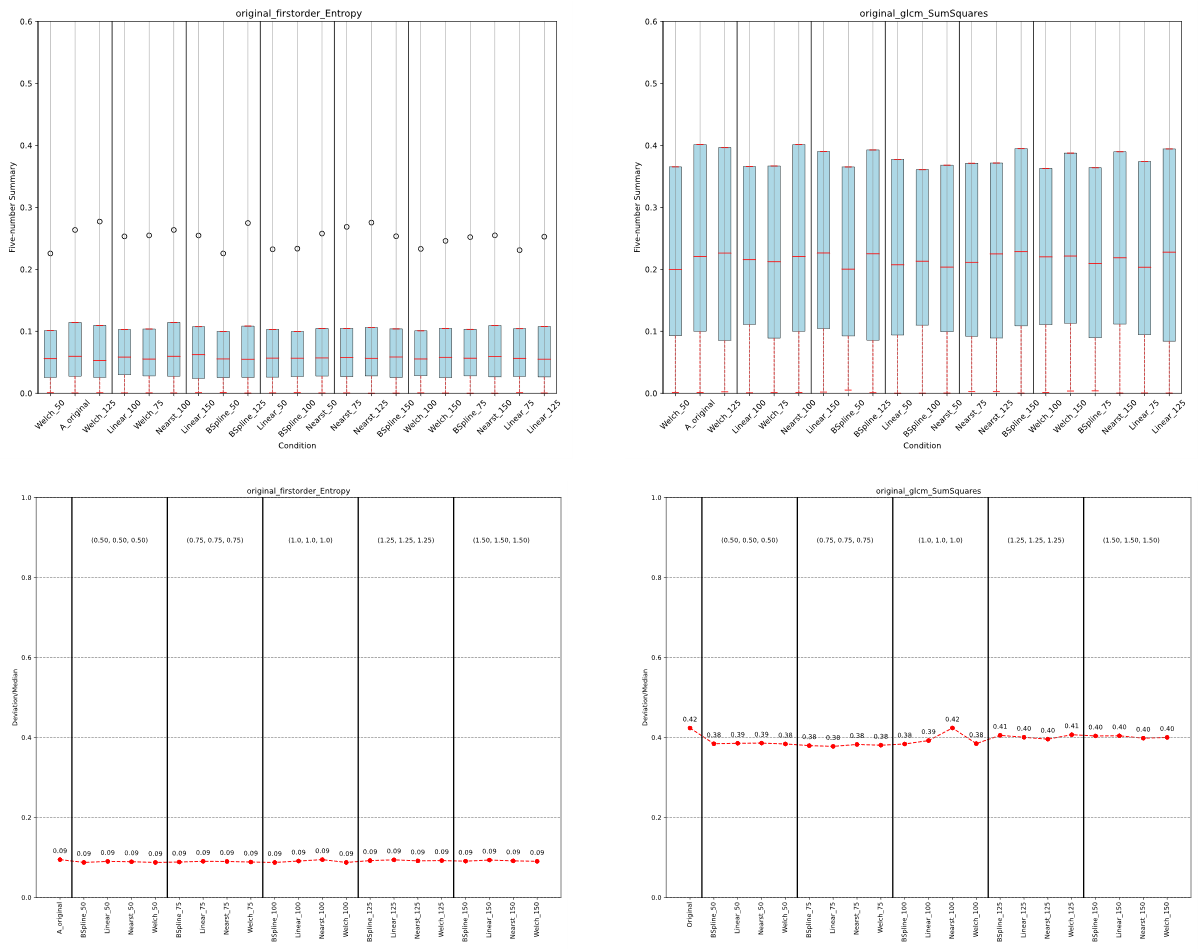


The horizontal axis represents the 21 imaging processing configures. The vertical axis represents the ratio of feature value standard deviation to median. The left plot is original_firstorder_entropy; the right is original_glcm_sumsquares.

**Supplemental Figure 4:** Shapley Additive explanations plot: the impact of stable radiomics features for diagnosing major coronary segments stenosis in the SVM, KNN, Naïve Bayes, MLP, Gradient Boosting, and Light BGM.


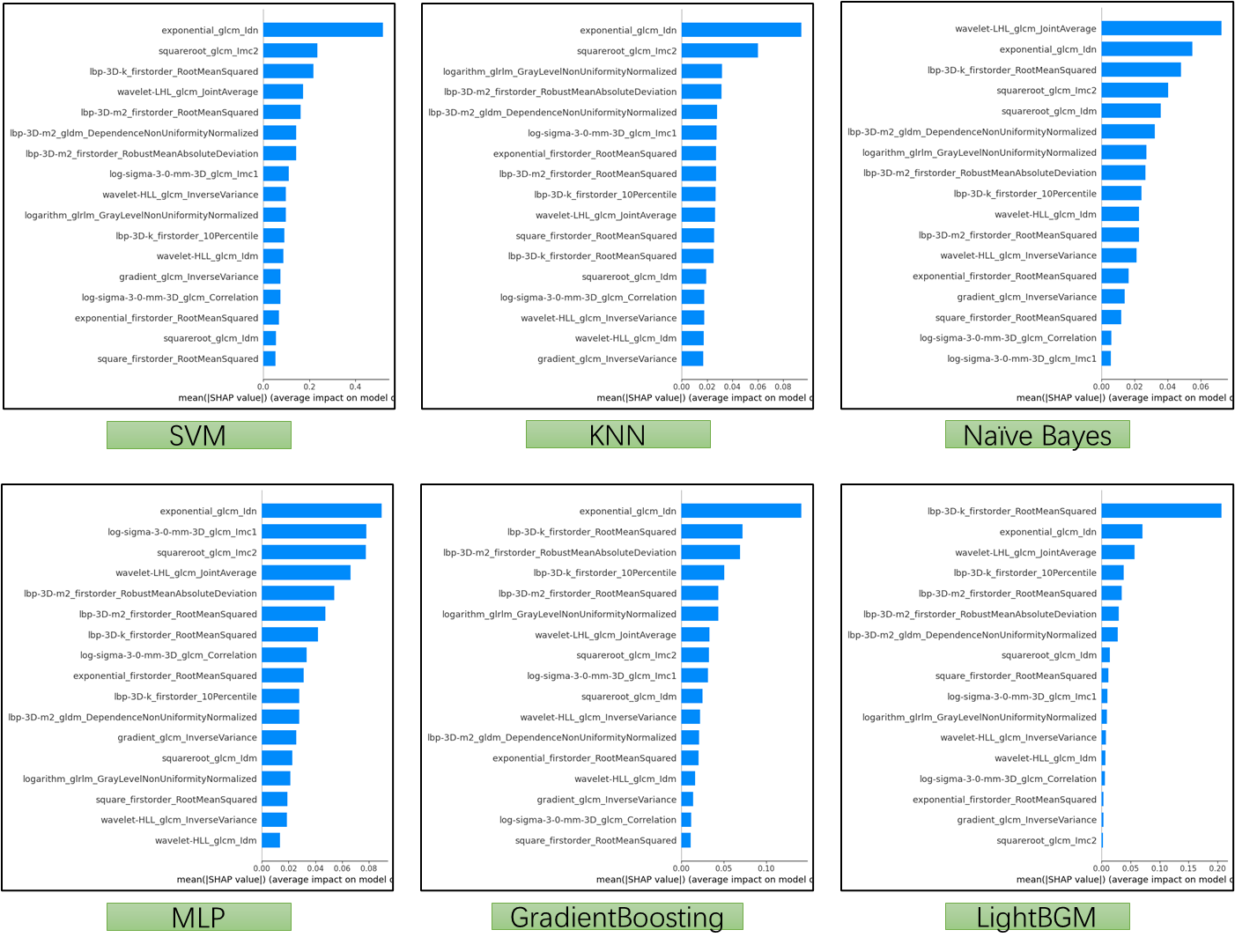


The bar plot represents the importance of the key features and their overall contribution to the models’ prediction outcome.

**Supplemental Figure 5:** Comparison of model accuracy across feature sets.


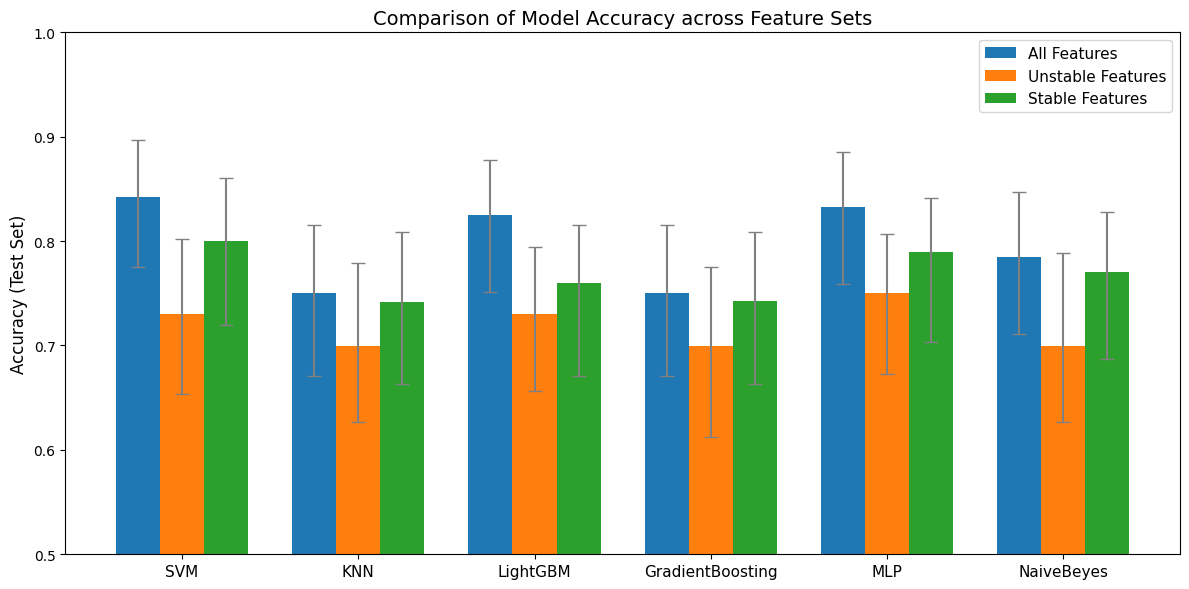


**Supplemental Figure 6:** Forest plot of the associations between key imaging features and myocardial infarction based on univariable Cox proportional hazards regression.


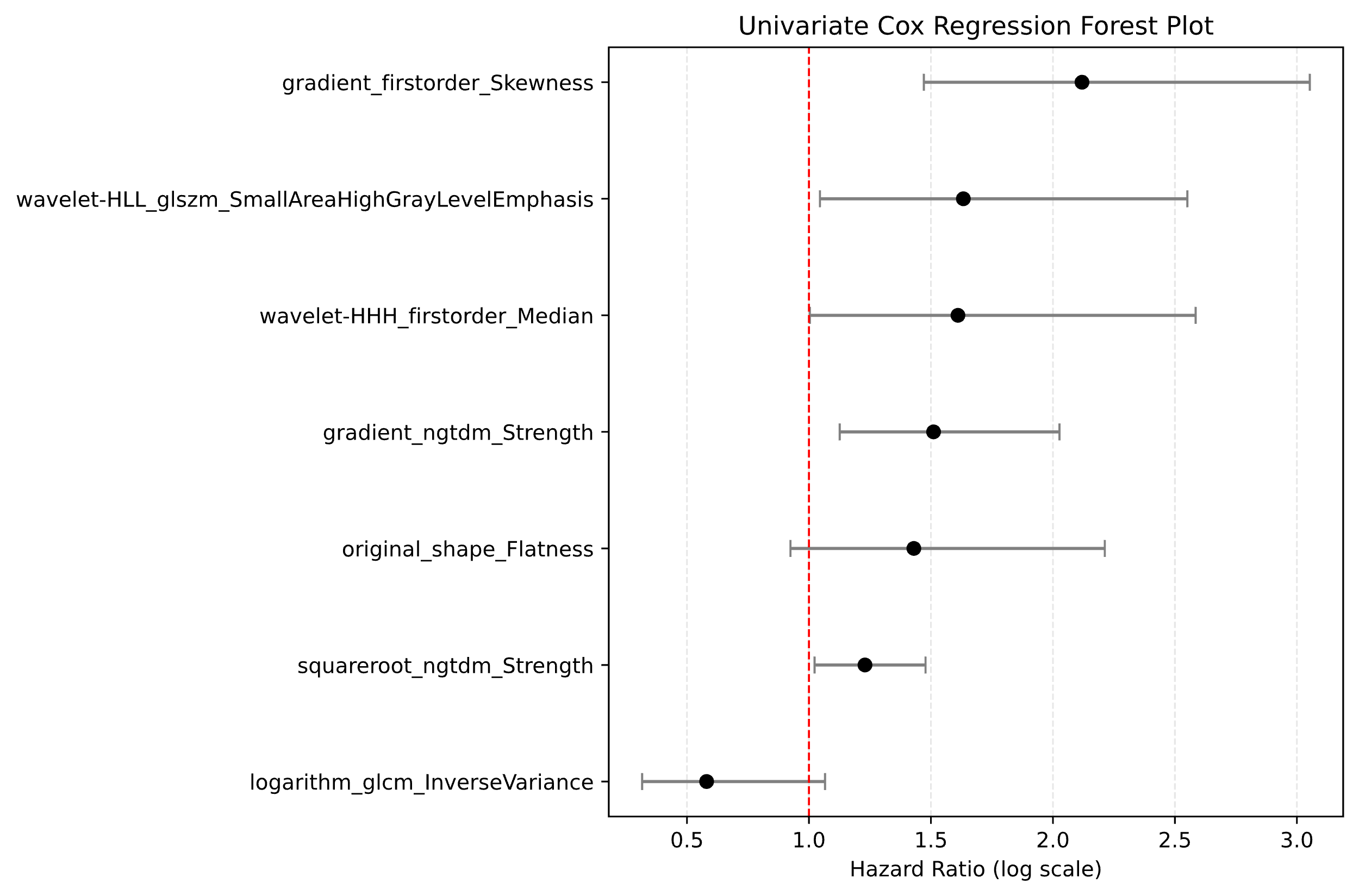


**Supplemental Figure 7:** Correlation between selected radiomic features and coronary artery calcium score (CACS).


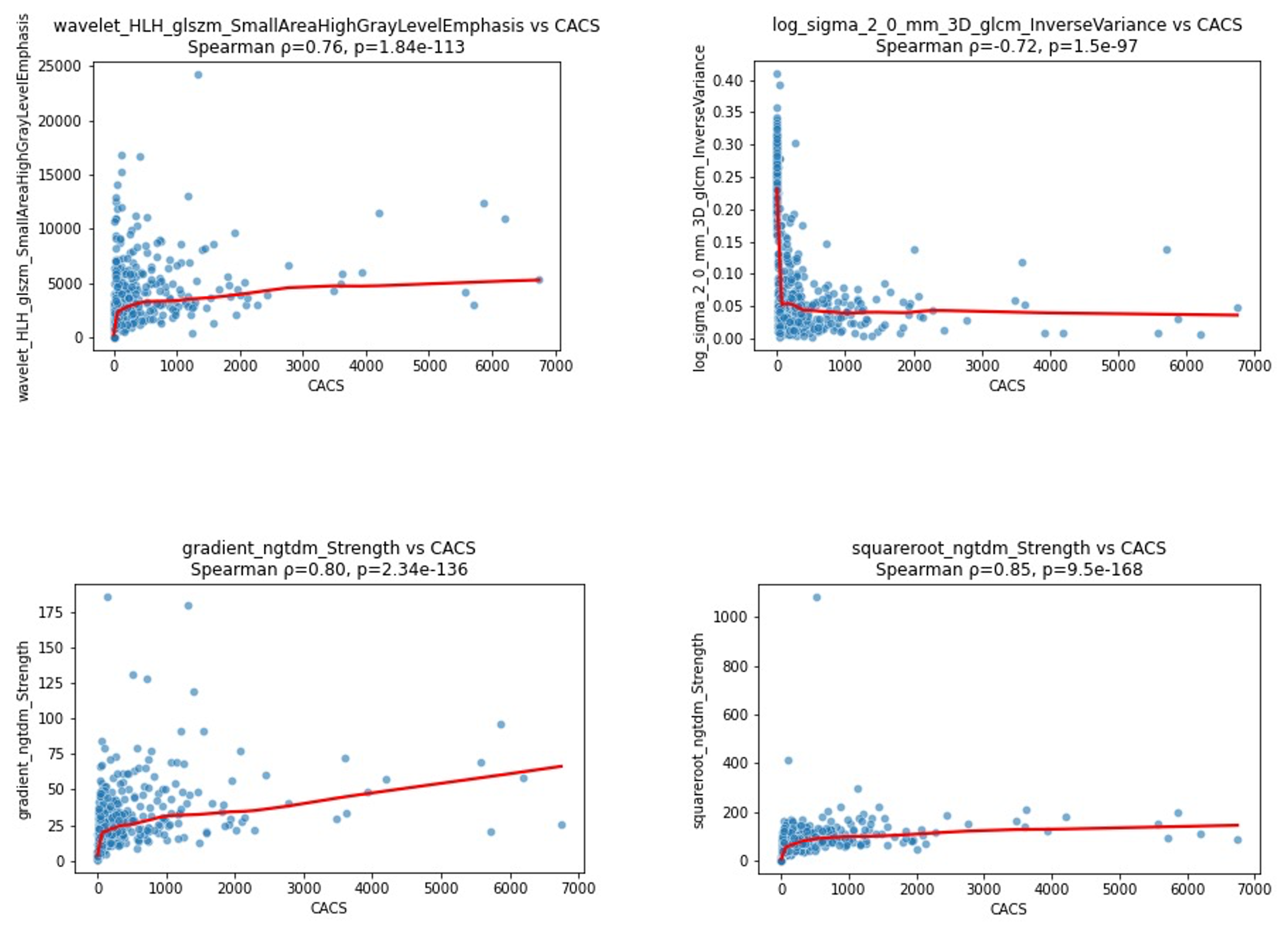


Scatter plots show the relationships between four representative radiomic features and CACS, along with corresponding Spearman correlation coefficients and p-values. The red line represents a fitted local polynomial regression (LOESS) trend.

**Supplemental Figure 8:** External Validation of Coronary Calcium Quantification Performance.


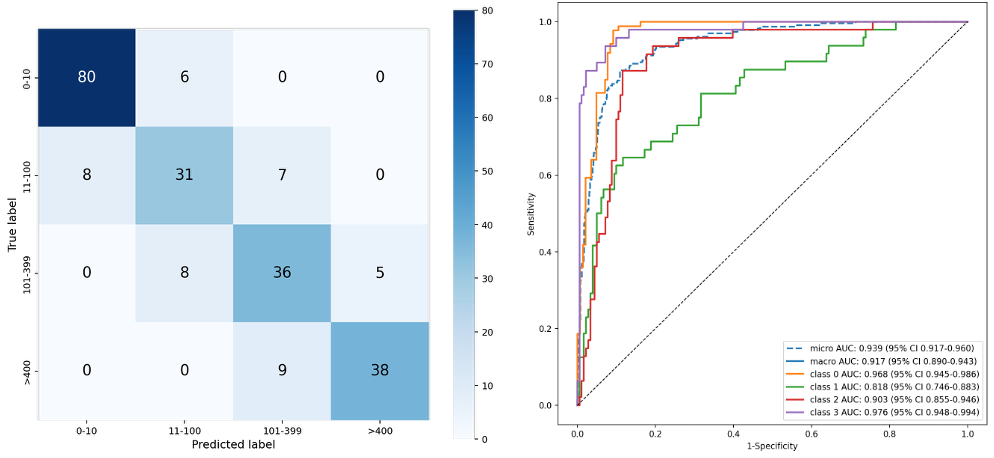


8. Radiomics feature categories

shape, first-order, Grey Level Co-occurrence Matrix features (GLCM), Grey Level Dependence Matrix (GLDM), Grey Level Run Length Matrix (GLRLM), Grey Level Size Zone Matrix (GLSZM) and Neighbouring Grey Tone Difference Matrix Features (NGTDM).

9. Stable feature selection

we considered the variance in the feature values across participants, which was quantified using the normalised standard deviation in addition to:

$\sigma_{n}=\frac{\sigma(f)}{|\mathrm{median}(f)|}$ (1)

Here, σ represents the standard deviation of *f*, the value of the feature across the healthy cohort. This extra measure was employed to identify features that exhibited very small (or nearly zero) variance under specific conditions. The list of stable features is in the Appendix 2.

**Supplemental** Appendix 2: The list of stable features

**Supplemental** Appendix 3: p-values for inter-group comparisons
